# Gas fields and large shallow seismogenic reverse faults are anticorrelated

**DOI:** 10.1038/s41598-022-05732-8

**Published:** 2022-02-03

**Authors:** G. Valensise, F. Donda, A. Tamaro, G. Rosset, S. Parolai

**Affiliations:** 1 Istituto Nazionale di Geofisica e Vulcanologia–INGV, Rome, Italy; 2 Istituto Nazionale di Oceanografia e di Geofisica Sperimentale–OGS, Trieste, Italy

**Keywords:** Natural hazards, Energy science and technology, Geophysics, Seismology

## Abstract

We investigated the spatial relationships among 18 known seismogenic faults and 1651 wells drilled for gas exploitation in the main hydrocarbon province of northern-central Italy, a unique dataset worldwide. We adopted a GIS approach and a robust statistical technique, and found a significant anticorrelation between the location of productive wells and of the considered seismogenic faults, which are often overlain or encircled by unproductive wells. Our observations suggest that (a) earthquake ruptures encompassing much of the upper crust may cause gas to be lost to the atmosphere over geological time, and that (b) reservoirs underlain by smaller or aseismic faults are more likely to be intact. These findings, which are of inherently global relevance, have crucial implications for future hydrocarbon exploitation, for assessing the seismic–aseismic behaviour of large reverse faults, and for the public acceptance of underground energy and CO_2_ storage facilities—a pillar of future low carbon energy systems—in tectonically active areas.

## Introduction

Thrust faulting earthquakes are an inherent occurrence in hydrocarbon-bearing active regions dominated by crustal shortening: they may be events generated by regional-scale tectonic processes, but also events triggered at all stages of hydrocarbon exploitation activities^1–4^. In most active hydrocarbon-bearing regions worldwide, however, earthquakes are often irregularly scattered across relatively homogeneous tectonic trends. For instance, a thorough investigation of the seismicity of the Zagros region of southern Iran^5^, one of the largest oil and gas reserves worldwide, revealed not only that thrust faulting earthquakes are less frequent than strike-slip events, despite the characteristic and actively deforming fold and thrust structure of the region, but also that the observed seismicity accounts for a small fraction of the range shortening estimated from GPS, InSAR and other lines of evidence. In the frictional regime, the fraction of fault slip that is released in earthquakes is generally referred to as *seismic coupling*, or *c*, a dimensionless parameter originally introduced based on observations of great earthquakes in the circum-Pacific belt^6^. Based on an investigation of the compressional domains encircling the Italian peninsula, most of which host important oil and gas reservoirs, *c* is about 50%, half than that estimated for the extensional domains straddling the Apennines chain^7^. Rather than being evidence for an episodically aseismic behaviour of the large thrust faults occurring beneath Italy's hydrocarbon reservoirs, this suggests that about one out of two of such faults slips consistently in an aseismic fashion. Moreover, stick–slip behaviour was suggested to occur only where previous tectonic histories caused high-stiffness rocks (e.g. Triassic and Jurassic limestones and dolostones) to be uplifted and brought in contact across the fault plane, up to the characteristic 3–10 km depth of local upper crustal thrust faults^7^.

This study explores the spatial distribution of productive and unproductive gas fields in relation with the occurrence of large active and potentially seismogenic faults, capable of generating M_w_ 5.5 + earthquakes (allowing for a +/− 0.2 uncertainty), that is the lower bound adopted by the DISS database (see “Methods” section). To this end, we use well locations as a proxy for the location of gas fields to assess their spatial correlation with fault locations. Notice that for simplicity we use the term “productive” for those wells whose logs report the occurrence of gas within the sedimentary succession, without necessarily implying that they have been commercially exploited; conversely, we use “unproductive” for wells that did not encounter any gas.

**Figure 1:**
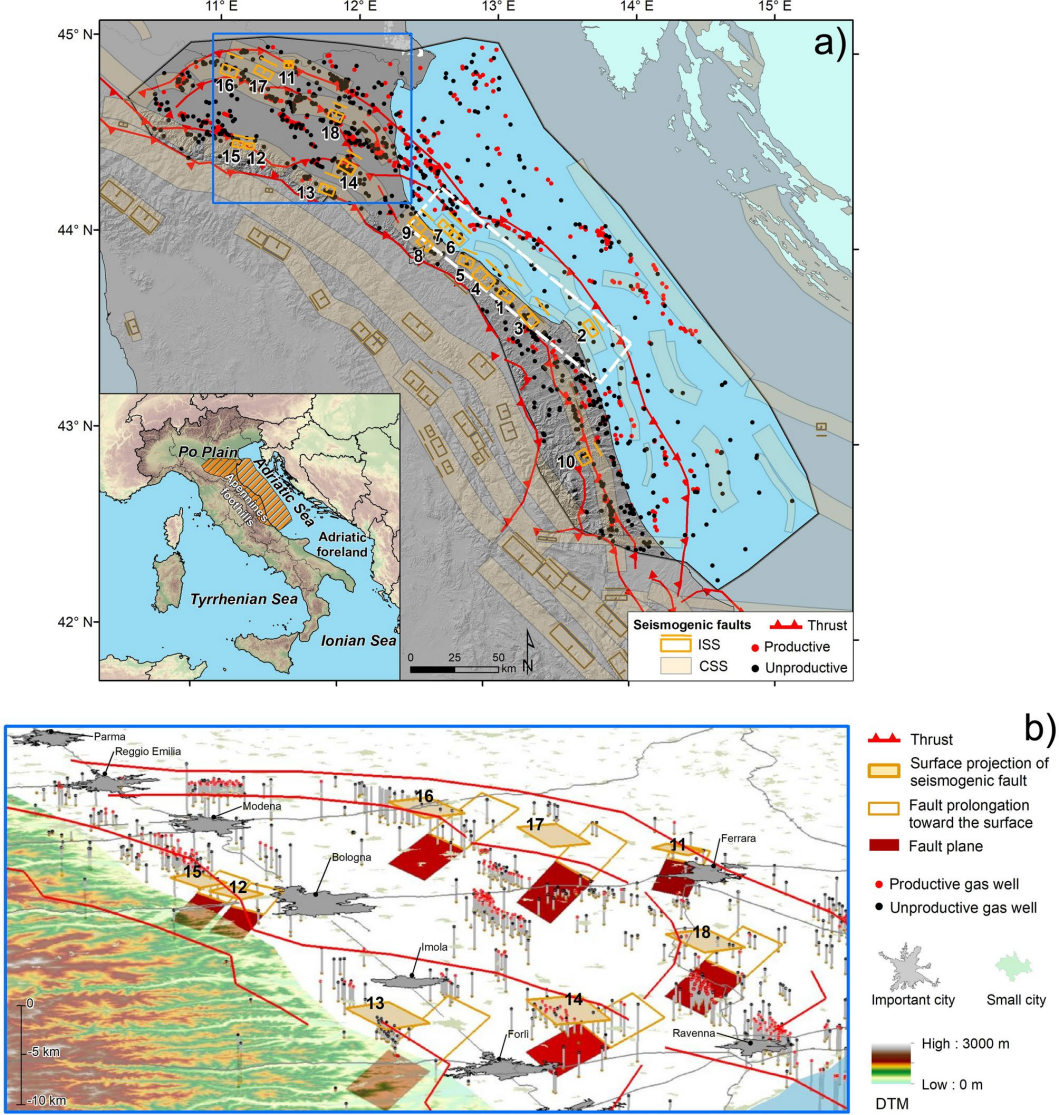
Overview of the study area, showing the location of selected gas wells and seismogenic faults. (**a**) The study area, shown by a black polygon, was delineated following the structural scheme proposed by Mantovani et al.^17^. Gas reservoirs are selected from the ViDEPI database and lie mostly within the external foothill belt between the leading thrusts of the internal zone and the outermost thrusts in the Adriatic foreland basin. The empty boxes are the surface projections of Individual Seismogenic Sources from the DISS database: the 18 faults selected for this study are shown in orange, whereas all others are shown in brown. The elongated light brown polygons are Composite Seismogenic Sources from the DISS database. The blue box shows the location of panel (**b**). The white dashed rectangle indicates a 120 km-long coastal strip where seismic hazard is consistently higher than in structurally similar, adjacent areas of the Italian peninsula, but where there exist only fewer-than-average unproductive wells and virtually no productive wells (see text). Please refer to the “Methods” section for further details. The figure was drawn using ArcGis 10.4.1 for Desktop/ArcMap 10.4.1.5686 by ESRI (http://www.esri.com). (**b**) Details of the geometrical calculations conducted over the northwesternmost portion of our study area. The solid orange boxes represent the surface projection of the selected faults (same as in panel **a**), whose full 3D extent is shown by the red patches, which extend from the minimum to the maximum depth of seismogenic faulting reported in the DISS database (see “Methods” section). The empty orange boxes show the fault prolongation toward the surface. The vertical lines show the location and depth of the investigated gas wells, allowing the reader to appreciate their actual 3D spatial relationships with the seismogenic faults. The figure was drawn using ArcGis 10.4.1 for Desktop/ArcScene 10.4.1.5686 by ESRI (http://www.esri.com).

Our study area spreads over a width of about 100 km from the inner portions of the Italian Apennines fold and thrust belt to the associated foredeep, and over a distance of about 300 km from the western Po Plain to the central Adriatic Sea (Fig. 1). The Apennines are a northeastern-verging portion of the peri-Mediterranean orogenic belt, formed as a consequence of the convergence and collision between the European and African plates since the Late Cretaceous^8^. They developed in Neogene and Quaternary times and currently comprise both an extensional domain straddling the range axis, and a compressional domain running along its Adriatic margin^9–12^. More specifically, the northern and central Apennines hydrocarbon province is a result of the progressive thrusting and folding, hosting hydrocarbon reservoirs at the core of growing anticlines/antiforms. The whole Italian peninsula is one of the richest hydrocarbon-producing regions in southern Europe^13,14^. Major gas fields lie parallel to the structural trends, and structural traps usually occur all throughout the thrust belt, most commonly along its external boundary, in the adjacent foredeep basin, and in the Adriatic foreland (Fig. 1). They concentrate in the Po Plain and in the northern and central Adriatic Basin, which are the targets of our investigation. Oil fields occur mostly in the western Po Plain, in the southern Apennines and in Sicily^15,16^.

Most of the Italian gas originates in Northern Adriatic reservoirs and is associated with two main source rock types. The older one is thermogenic gas-prone, the main gas pools being found in turbiditic sands occurring in the Apennines foothills^16^, whereas the younger one produces biogenic gas hosted in the outer Plio-Pleistocene foredeep domain and feeds the most important fields^14^. Biogenic gas is trapped in the highly efficient Plio-Pleistocene turbidite systems of the Apennines foredeep^18^ and within synsedimentary traps on both the inner and outer flanks of the folds^19^.

The occurrence of the 20 and 29 May 2012 thrust faulting earthquakes in the southern Po Plain (M_w_ 6.1 and 5.9, respectively) prompted a debate on the potential triggering role of hydrocarbon exploitation^20^. Mucciarelli and coworkers^21^ investigated the 2D spatial relationships between known seismogenic sources and the distribution of 455 gas fields in a ∼ 10,000 km^2^ portion of the central-southern Po Plain, and proposed the existence of an anticorrelation between productive reservoirs and the presence of relatively large seismogenic faults, i.e. faults capable of Mw 5.5 + earthquakes.

We aim at substantiating these hypotheses and results by investigating a four times larger—and unique, to our knowledge—sample of gas wells, occurring over a five times larger area, and a twofold number of seismogenic faults; we analysed them using a more detailed metric based on fully 3D distances between wells and faults and through a more robust statistical approach.

## Results

We explored in a GIS-environment the spatial relationships among 1651 wells drilled for gas exploitation and 18 known seismogenic faults (Fig. 1a and Table 1: see also the “Methods” section) occurring over an area of over 50,000 km^2^. We first calculated both minimum *planar* (2D) distances, from the well head to the closest point of a 2.0 km buffer drawn around the surface projection of each seismogenic fault, and minimum *three-dimensional* (3D) distances, from the well bottom to the closest point on the actual fault plane. The 2.0 km size of the buffer zone corresponds to 20% of the average length of the selected ISSs: we assumed it as a reasonable measure of the variability in the location of the rupture plane, in the absence of firmer constraints on the actual uncertainty.

**Table 1:** Main parameters of Individual Seismogenic Sources falling within our study area.

Source ID	DISS source ID	DISS source name	Min depth (km)	Max depth (km)	Fault dip (°)	Associated earthquake	Mw	Productive gas wells	Unproductive gas wells
1	ITIS024	Mondolfo	4.0	7.0	30	1 Feb 1924	5.3	–	4
2	ITIS029	Conero offshore	2.5	6.4	40	23 Dec 1690	5.4	–	1
3	ITIS030	Senigallia	4.0	7.5	30	30 Oct 1930	5.8	–	5
4	ITIS031	Fano Ardizio	3.0	7.0	30	Unknown	–	–	–
5	ITIS032	Pesaro S. Bartolo	2.5	5.9	35	Unknown	–	–	1
6	ITIS033	Rimini offshore south	3.0	5.5	30	16 Aug 1916	5.7	–	2
7	ITIS034	Rimini offshore	3.0	5.5	30	17 May 1916	5.8	–	1
8	ITIS035	Rimini	3	6.0	30	25 Dec 1786	5.6	–	1
9	ITIS036	Val Marecchia	3.0	6.0	30	17 Mar 1875	5.8	–	–
10	ITIS070	Offida	4.5	8.7	35	10 Mar 1943	5.8	1	3
11	ITIS090	Ferrara	1.4	4.5	50	17 Nov 1570	5.5	–	2
12	ITIS091	Casalecchio di Reno	2.0	4.2	35	03 Jan 1505	5.6	–	–
13	ITIS093	Faenza	4.5	7.8	35	04 Apr 1781	5.9	–	11
14	ITIS100	Bagnacavallo	2.5	5.0	25	11 Apr 1688	5.8	16	9
15	ITIS103	Crespellano	2.0	4.5	35	20 Apr 1929	5.4	–	–
16	ITIS107	Mirandola	4.0	7.0	30	29 May 2012	5.9	–	3
17	ITIS134	Finale Emilia	4.0	8.4	43	20 May 2012	6.1	–	–
18	ITIS141	Argenta	3.0	6.3	35	19 Mar 1624	5.4	1	6
				Wells falling within ISSs				18	49
				Total within study area				831	820
				Grand total				1651	

Summary of the seismogenic faults occurring in the study area, showing also the number of wells falling within the surface projection of each source (see the “Methods” section).

All earthquake magnitudes (M_w_) are from the CFTI5Med catalogue (http://storing.ingv.it/cfti/cfti5/), except for the 20 and 29 May 2012 events, whose magnitude is from the CPTI15 catalogue (https://emidius.mi.ingv.it/CPTI15-DBMI15/).

We then used the Weight of Evidence method (WofE^22^), a bivariate statistical analysis based on the Bayesian probability framework, to detect any statistically significant spatial correlation/anticorrelation between faults and productive/unproductive gas fields (Fig. 1b: see the “Methods” section for details). A positive C implies a good spatial correlation (the higher the value, the larger the correlation) between the well location and the fault plane; a C around 0 indicates a non-significant correlation; a negative C indicates absence of correlation. The Studentised contrast C/S(C), where S(C) is the standard deviation of C, provides insight into the statistical significance of the results (please refer to the “Methods” section for the other definitions used in the following).

### 2D distances

**Figure 2:**
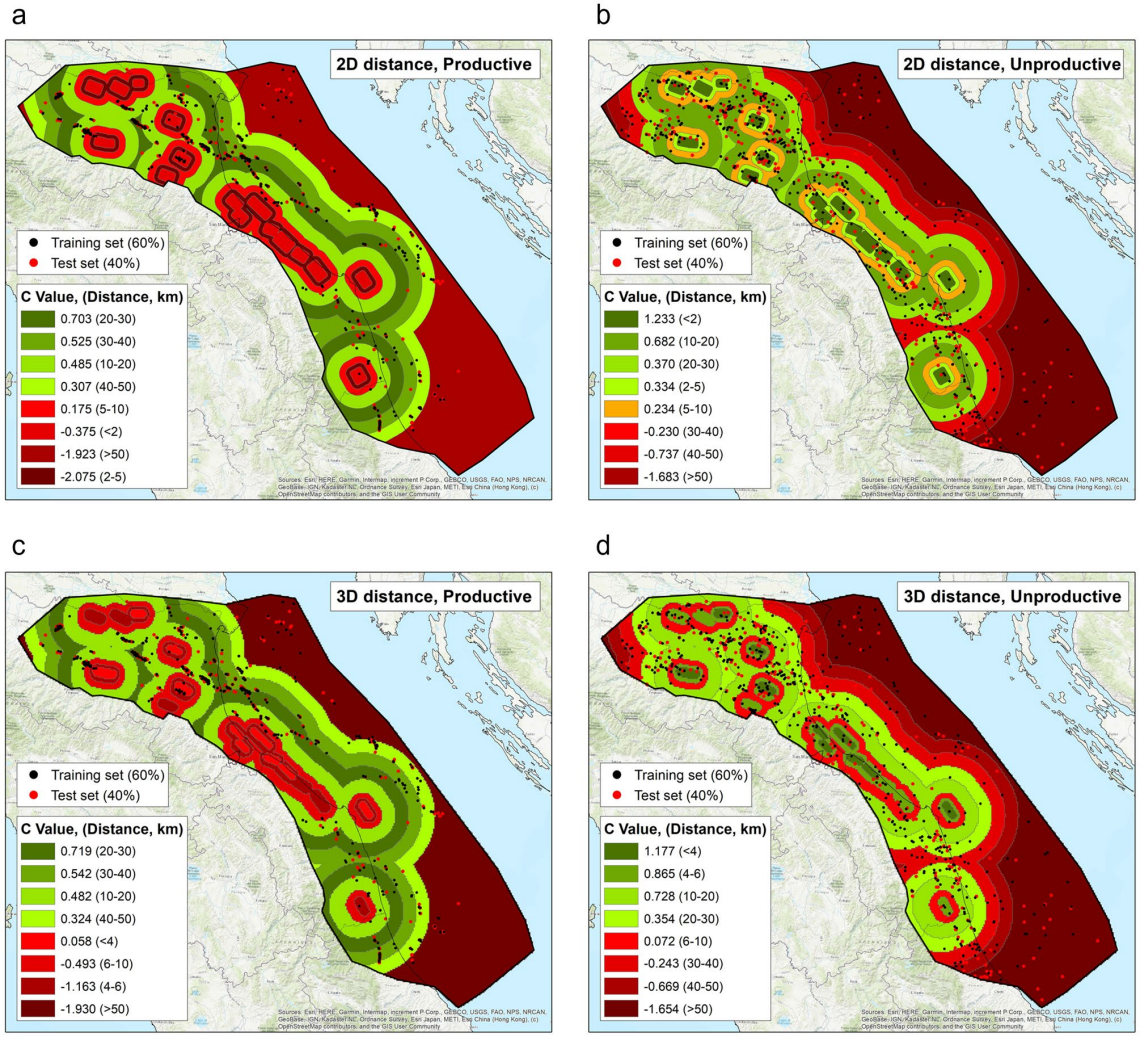
Distribution of C values for the 2D and 3D distance cases. Distribution of C values for the 2D (**a**, **b**) and 3D (**c**, **d**) distance cases, for productive (left) and unproductive wells (right), respectively. The figure was drawn using ArcGis 10.4.1 for Desktop/ArcMap 10.4.1.5686 by ESRI (http://www.esri.com).

Table 2a and Fig. 2a, b summarise the results obtained using the *training dataset* for the eight adopted distance bins in the 2D distance case.

**Table 2:** Summary of the results obtained through the Weight of Evidence test.

Distance bin (km)	Contrast weight (C)	C/S (C)	Total wells (training dataset)	Total wells (training dataset %)	Total wells (testing dataset)	Total wells (testing dataset %)
**(a)**						
2D distance—productive wells						
< 2	− 0.375	− 1.385	14	2.8	7	2.1
2–5	− 2.075	− 3.583	3	0.6	5	1.5
5–10	0.175	1.172	50	10.0	19	5.7
10–20	0.485	4.786	133	26.7	86	25.9
20–30	0.703	6.715	120	24.0	95	28.6
30–40	0.525	4.467	88	17.6	53	16.0
40–50	0.307	2.246	61	12.2	41	12.3
> 50	− 1.923	− 10.213	30	6.0	26	7.8
2D distance—unproductive wells						
< 2	1.233	9.077	62	12.6	30	9.1
2–5	0.334	1.800	31	6.3	22	6.7
5–10	0.234	1.597	52	10.6	39	11.9
10–20	0.682	6.979	151	30.7	96	29.3
20–30	0.370	3.190	91	18.5	59	18.0
30–40	− 0.230	− 1.470	45	9.1	34	10.4
40–50	− 0.737	− 3.450	23	4.7	16	4.9
> 50	− 1.683	− 9.847	37	7.5	32	9.8
**(b)**						
3D distance—productive wells						
< 4	0.058	0.18	10	2.0	4	1.2
4–6	− 1.163	− 3.06	7	1.4	9	2.7
6–10	− 0.493	− 2.494	27	5.4	87	26.2
10–20	0.482	4.865	143	28.7	103	31.0
20–30	0.719	7.018	128	25.7	53	16.0
30–40	0.542	4.694	92	18.4	41	12.3
40–50	0.324	2.402	63	12.6	8	2.4
> 50	− 1.930	− 10.088	29	5.8	27	8.1
3D distance—unproductive wells						
< 4	1.177	6.150	29	5.9	10	3.0
4–6	0.865	5.693	48	9.8	25	7.6
6–10	0.072	0.459	45	9.1	45	13.7
10–20	0.728	7.645	167	33.9	101	30.8
20–30	0.354	3.096	95	19.3	63	19.2
30–40	− 0.243	− 1.568	46	9.3	35	10.7
40–50	− 0.669	− 3.260	25	5.1	17	5.2
> 50	− 1.654	− 9.676	37	7.5	32	9.8

Summary of the results obtained for the 2D (a) and 3D (b) distance scenarios, respectively for productive and unproductive wells.

For productive wells, C values are negative for distances < 5 km (down to − 2.075); they then increase, reaching a maximum in the 20–30 km bin, and become negative again for distances > 50 km. These results indicate that productive wells generally locate rather far from seismogenic faults but not beyond 50 km, a distance that may exceed the half-width of the hydrocarbon province investigated in this work.

In marked contrast, for unproductive wells the largest C values (1.233) and the largest Studentised contrast (9.077) are found for the first distance bin (0–2 km). C drops quickly for increasing distance ranges, becoming negative (− 1.683: C/S(C) − 9.847) for a distance > 50 km. These results indicate that the wells falling very close to a seismogenic fault are likely to be unproductive.

### 3D distances

Table 2b and Fig. 2c, d summarise the results obtained using the *training dataset* for the eight adopted distance bins in the 3D case.

The results we obtained for productive wells indicate a non-significant correlation (C = 0.058) for the shortest distance bin (< 4 km); the ratio between the contrast weight and the standard deviation (0.180) for the closest bin of the 3D distances, however, indicates that an interpretation of the results should not be attempted. C is negative in the 4–6 km and 6–10 km bins (− 1.163 and − 0.493) and becomes positive C for distances > 10 km, implying that productive wells are generally drilled > 10 km from seismogenic faults. For distances > 50 km we found a negative C (− 1.930), as in the 2D case. The results suggest no statistical correlation, or a negative correlation, between the location of the productive wells and that of seismogenic faults.

As for unproductive wells, the largest C (1.177) is found for the shortest distance bin (< 4 km). C then decreases up to a 10 km distance, increases again in the distance range 10–20 km, and drops beyond 50 km, becoming fully negative.

### Success-rate curves

**Figure 3:**
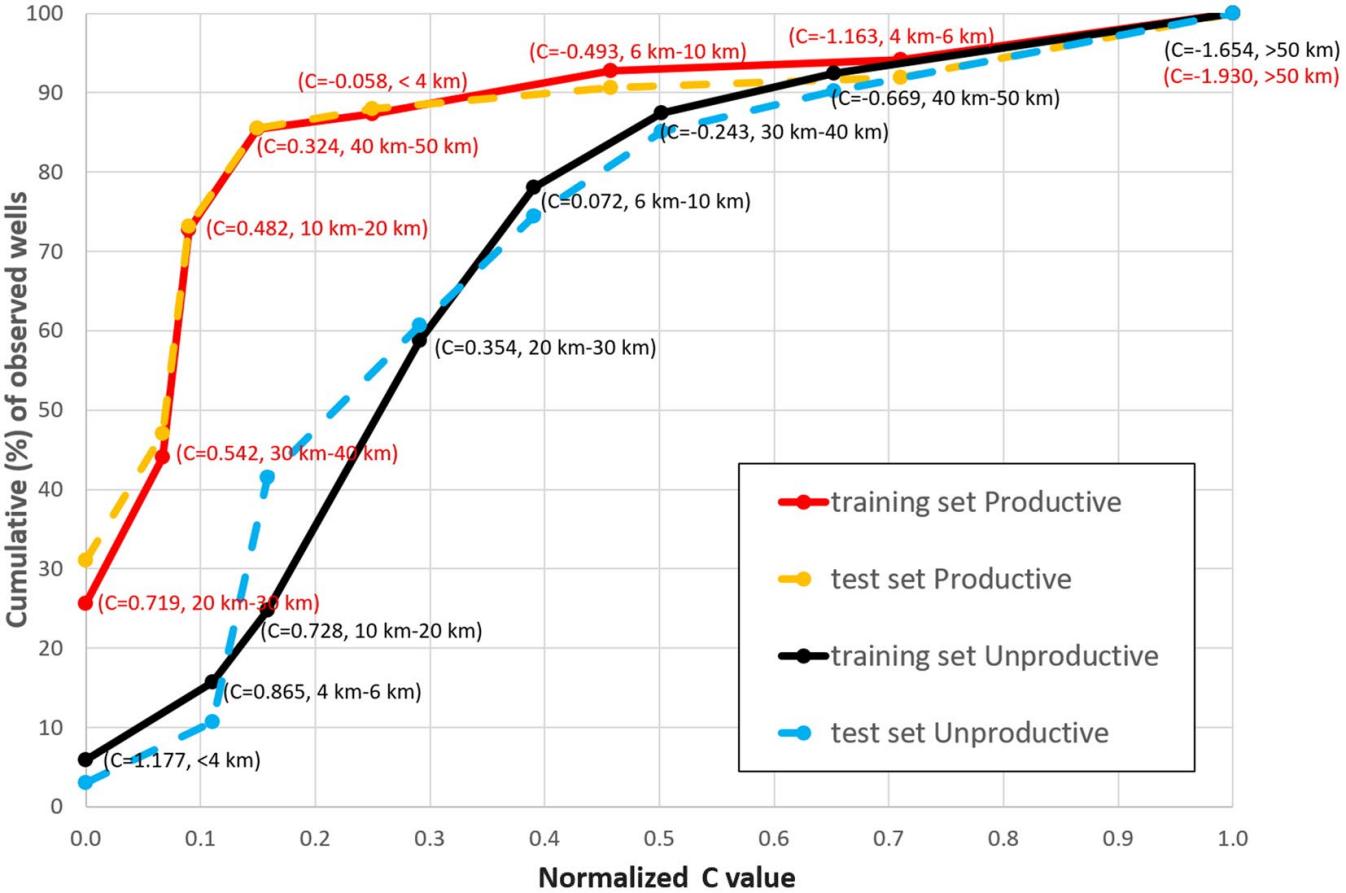
Success-rate curves and prediction-rate curves for the 3D scenario. Success-rate curves (in red for productive and black for unproductive) and prediction-rate curves (in orange for productive and blue for unproductive) of C distributions calculated for the 3D distance scheme.

As detailed in the “Methods” section, we calculated the success-rate curve using our *training dataset*, then used the *testing dataset* for estimating the prediction-rate curve (Fig. 3).

In the 2D case, 80.3% and 78.3% of the data in the *training dataset* are found in the 20% and 35% largest C bins, respectively for productive and unproductive wells. The Area Under the Curve (AUC: see the “Methods” section for its exact definition) estimated for the success-rate curve is very good for productive wells (92.5%), and good for unproductive wells (80.0%).

In the 3D case, 85.5% and 68.8% of the data in the *training dataset* are found in the 20% and 30% of the largest C bins, respectively for productive and unproductive wells. The AUC estimated for the success rate curve is very good for productive wells (90.2%), and good for unproductive wells (77.2%).

In both the 2D and 3D cases the AUCs calculated from the prediction-rate curves for the *testing dataset* are similar to those estimated based on the *training dataset*, suggesting that the model performs well in predicting the typology of the productive/unproductive nature of a well depending on its 2D/3D distance from a seismogenic fault.

The results of our statistical analysis show a clear anticorrelation between the spatial distribution of productive wells and of seismogenic faults, whereas the correlation between unproductive wells and faults is more tenuous, albeit still statistically significant. In our opinion this circumstances reflect a specific characteristic of the data that will be elucidated later on.

### Overview of the results

Unlike other applications of the WofE method, in this work we had to deal with two widely independent sources of uncertainty: (a) the incompleteness of the dataset of gas reservoirs, mainly concerning their depth, and (b) the inevitably limited and fragmentary knowledge on seismogenic faults; their location and geometry generally rely on good quality geological and exploration seismology data, but their activity is documented by earthquakes that are difficult to assign to a specific causative fault as they occurred up to four centuries ago.

Nevertheless, our analyses document a statistically significant relationship between the location of productive/unproductive gas wells and the occurrence of seismogenic faults deemed capable of M_w_ 5.5 + earthquakes. More specifically, the WofE shows that the highest spatial correlation between the vast majority of productive wells and the selected seismogenic faults is obtained for a 10–50 distance: in 402 out of 499 cases (80%) for the 2D distance scenario, and in 405 out of 499 (81%) for the 3D scenario. These figures imply that the existence of a productive well above an identified seismogenic source is a statistically rare occurrence throughout our study region. Conversely, unproductive wells show a high spatial correlation with the location of seismogenic faults, although—somewhat unexpectedly—the results of the WofE method for 2D distances are more robust than those obtained using 3D distances. These results do support our working hypotheses, but their interpretation requires a closer inspection of the geographical distribution of the data.

In summary, productive wells generally locate away from large seismogenic faults, whereas unproductive wells are often associated with them. The only significant exceptions reported for the 2D scenario are 14 productive wells lying in the class 0–2 km (Table 2a, *training dataset* only), 11 of which are located within the surface projection of the individual source ITIS100 Bagnacavallo, two fall within the ITIS141 Argenta, and one falls within the ITIS070 Offida (Fig. 1, Table 1). In the 3D scenario we find only 10 wells falling within 4 km of the fault.

## Discussion

### Statistical significance of the data and of the results

Our elaborations show that for productive wells, large C values are obtained only for relatively large well-to-fault distances, whereas unproductive wells correlate with seismogenic faults primarily for short distance ranges, but exhibit relatively high C values also for more distant ranges, up to 30 km. Also, productive wells tend to cluster in space, whereas unproductive wells exhibit a more scattered distribution over the whole study area (this is clearly seen in Figs. 1, 2 and S3–S6). These two conditions are a clear indication that both productive and unproductive wells may not provide a totally reliable portrait of their own distribution.

Why does this happen? Generally, once a newly drilled well is found productive, additional wells are drilled around it; conversely, if that well is found unproductive, the exploration generally stops, as further activity nearby is unlikely to be economically attractive. The result of this quite understandable imbalance is that, at least in our study area, the exploration has carefully circumvented large areas that in principle were suitable for hydrocarbon accumulation, based on their geological setting, but had already shown to be unproductive. This is the case of the nearly 120 km-long coastal strip stretching from Rimini to Ancona, a region where seismic hazard is consistently higher than in structurally similar, adjacent areas (http://zonesismiche.mi.ingv.it/), but where there exist only a handful of unproductive wells and virtually no productive wells (Figs. 1, 2).

The combination of these circumstances shows (a) that the wells are not randomly distributed in space, as an ideal statistical analysis would require, and (b) that certain “extreme” areas, where the absence of productive reservoirs has been known for decades, are severely undersampled. Nevertheless, we maintain that this inevitable imbalance of our sample is at least partially compensated by its richness.

Our elaborations also show that productive gas wells—and therefore, major gas reservoirs—occasionally occur above a seismogenic fault. This departure from their expected behaviour could be related to local heterogeneities with respect to the predominant Plio-Pleistocene stratigraphic setting of the Apennines fold and thrust belt and foredeep. Such variations could indeed occur at a local scale, but the dataset we analysed reveals the nearly exclusive occurrence of Plio-Pleistocene gas-charged or sterile sandy-silty sedimentary successions^15,18,19^.

Aside from stratigraphic considerations, the occurrence of a gas reservoir above a seismogenic fault may simply indicate that the fault is poorly located, for example because it is deeper than assumed in the DISS database, or alternatively, that there is a flaw in the physical behaviour implied in the proposed anticorrelation between faults and productive wells. For ITIS100 and ITIS141 the first scenario is more likely, as these two sources are held responsible for earthquakes that occurred over three centuries ago; for ITIS070 one might consider that the productive well overlying it reaches a depth of 2.5 km, whereas the fault is assumed to extend between 4.5 and 8.7 km depth.

Since all fault data are inherently uncertain within a few km, and in consideration of the large number of data analysed in this work, we consider that the trend highlighted by the statistical analysis, and confirmed by the additional tests described in the “Methods” section and shown in the Supplementary Information, describes well a common behaviour. Individual outliers will of course deserve future, more focused investigations.

### Structural interpretation of the results

Having established the statistical significance of the results, the next question is: what relates earthquakes and hydrocarbon reservoirs? Unquestionably, the success or failure of hydrocarbon exploration in a fold and thrust belt is primarily controlled by the relative timing of source rock maturation, hydrocarbon migration and trap development. The outcomes of our study, however, strongly suggest that an equally crucial pre-requisite for the formation of an efficient gas reservoir is that the deposits overlying the reservoir-bearing formations be unaffected by active fractures and faults which might allow fluids to escape to the atmosphere. This condition is not warranted in earthquake-prone areas^21^, where sudden (seismogenic) fault slip may exceed the ductility of the sealing layers, causing brittle failure and fluid pressure breach.

**Figure 4:**
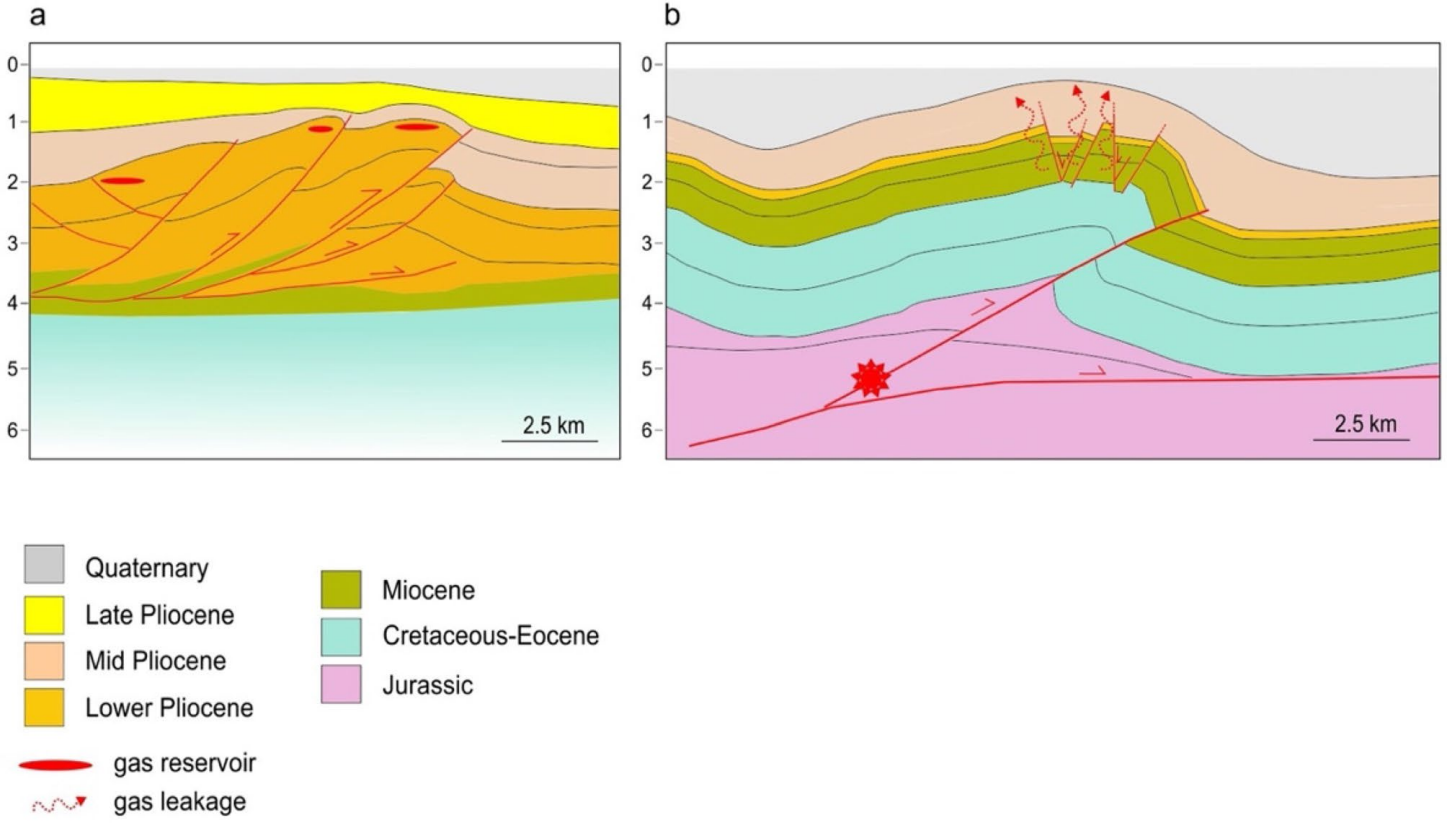
Schematic cross sections across the outermost fronts of the Apennines fold and thrust belt. (**a**) Small-scale anticlines hosting gas reservoirs (modified from Casero and Bigi^16^). (**b**) Larger-scale folds, driven by deeper and larger faults, which may generate relatively large earthquakes (Mw 5.5 + : red star; redrawn from Emami et al.^23^).

A close inspection of Fig. 1—and of the ample literature that lies behind it (see the Introduction)—shows that most productive reservoirs are hosted in small-scale anticlines (Fig. 4a), generated by faults that are shorter and narrower with respect to the deep and large faults driving long-wavelength folds that may generate significant earthquakes, and where gas is generally not found (Fig. 4b). In the DISS database the larger and positively seismogenic faults responsible for creating potentially large folds are listed as Individual Seismogenic Sources (ISSs). Smaller folds/reservoirs are either delineated as Composite Seismogenic Sources (CSSs), implying that they may generate M_w_ 5.5 + earthquakes but their ability to slip seismically vs. aseismically is currently unknown, or are not mapped at all, implying that they are considered just too small to generate M_w_ 5.5 + earthquakes.

Mucciarelli et al.^21^ contended that if the fault that controls the evolution of a potential hydrocarbon trap generates a significant earthquake, the integrity of the sealing horizons will inevitably be jeopardised. They also stressed that any subsequent gas loss is not caused by the earthquake shaking per se, but rather by the actual fault slip and its consequences. A M_w_ 5.5 + earthquake is necessarily the result of a few tens of cm of coseismic slip of a fault that may extend over a considerable thickness of the upper crust—generally between 2–3 and 8–10 km depth in our study area—and potentially up to the surface; larger events will cause larger slip over a bigger fault, whereas the slip associated with smaller events will simply be too small and too deep to affect the integrity of the sealing horizons.

Coseismic strain may propagate upward following different mechanisms, including the presence of overpressured fluids, leading to the creation or rejuvenation of fractures and faults that may immediately act as pathways for fluid escape. Such mechanisms are compatible with the limited distance we calculated between the location of unproductive wells and the location of known seismogenic faults—less than 2 km for the 2D case, less than 6 km for the 3D case (see Tables 2 and S2)—even taking into account the uncertainties discussed earlier. As we stated earlier, pushing our interpretations to areas where only earthquakes smaller than M_w_ 5.5 are expected would be inappropriate, as the coseismic strain caused by their causative faults would simply be too small to activate the mechanisms advocated in this work.

Notice that the crustal volume located at the tip of a thrust fault has long been known to be characterised by extensive fracturing, allowing for an efficient migration of fluids toward the surface^24–28^. The density of fractures and the wide range of their orientation is typical of the fault damage zone, a volume of intense rock deformation resulting from the initiation, propagation and build-up of slip along the main underlying fault^29–32^.

**Figure 5:**
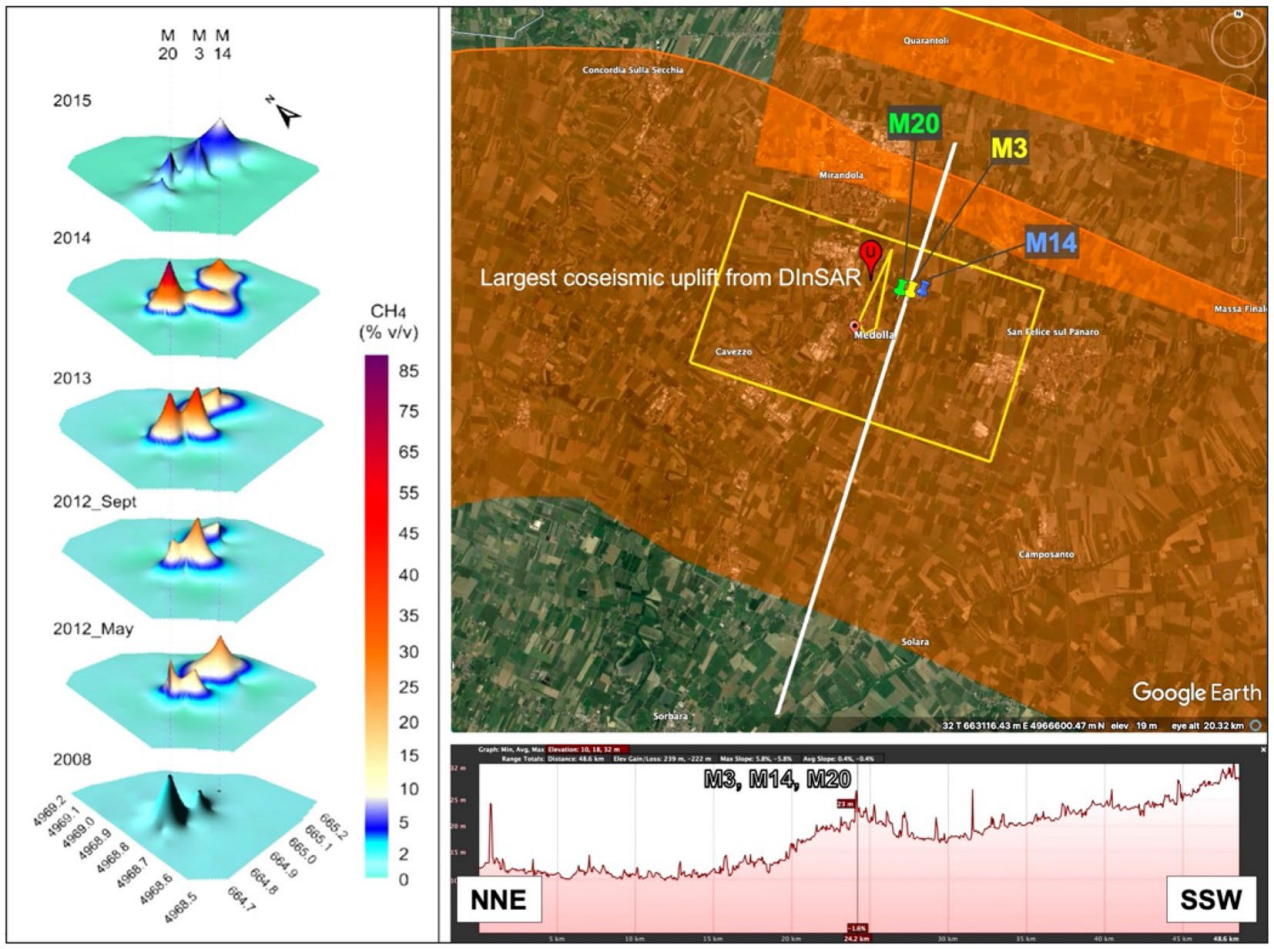
Location of the historically documented Medolla macroseeps. Summary of occurrences in the region of *Terre calde* (Warm grounds), a set of permanent macroseeps documented historically near Medolla (Modena, northern Italy). Their name relates to the continuous degassing, which eventually heated the ground causing anticipated melting of the snow cover in winter. Left panel): changes in the natural release of CH_4_ between 2008 and 2015 for the M3, M14 and M20 emissions^33^. Upper right panel): surface projection of the Individual Seismogenic Source responsible for the 29 May 2012 earthquake, shown in yellow, overlying Composite Seismogenic Sources, in orange (all from the DISS database). The image shows also the culmination of vertical dislocation detected by DInSAR interferometry following the 29 May 2012 Mirandola (Emilia) earthquake^34^, and the exact location of the M3, M14 and M20 emissions. Lower right panel): topographic profile of the area along a NNE-SSW trend (in light gray in the overlying map), showing that the emissions fall on a local altimetric culmination and in the area of largest coseismic uplift. Such mechanism of continuous degassing is most likely due to extrados faulting; a mechanism that has been shown to dominate in the northern Apennines, at the western end of our study area^35,36^, and may be common elsewhere. We contend that a similar mechanism might explain many of our observations over the entire study area, and over any similar areas around the globe. See text for further discussion. Base map and profile courtesy of Google Earth Pro v. 7.3.4.8248, 16 July 2021: map centred on Medolla (Modena, northern Italy), 32 T 663,975.81 m E, 4,968,157.45 m N, elevation 24 m, eye altitude 20.32 km, imagery date 8/18/2014–9/4/2020 (online). Available through:   https://earth.google.com/web/  (accessed 9 January 2022).

All of these processes are well described in the literature and have been extensively investigated with analogue and analytical models, but they have seldom been observed in the field. A notable exception is discussed by Sciarra et al.^33^, who investigated in detail the release of underground fluids, including CH_4_, following the 29 May 2012, M_w_ 5.9 earthquake near Mirandola (southern Po Plain). The event was generated by a thrust fault that lies beneath the Mirandola anticline and was assumed to be potentially seismogenic as early as 2000 in a prototype of the DISS database. At least six unproductive wells (Bignardi, Camurana, Cavone, Concordia, Medolla, San Biagio) lie on the surface projection of the fault, supporting the anticorrelation advocated in this work. The area located just northeast of the village of Medolla has been known at least since 1838 for the existence of gas macroseeps located exactly above the culmination of the buried Mirandola anticline (Fig. 5), as suggested by subsurface geology and topographic evidence and by the pattern of coseismic elevation changes following the 2012 earthquake^34^. The combination of this evidence with the data presented by Sciarra et al.^33^ suggests that these macroseeps are the surface evidence of extrados normal faults associated with the buried Mirandola anticline (Fig. 5).

Their CH_4_ flux measurements show that the sealing horizon of the local reservoir is partially efficient during the interseismic period, but fails catastrophically following an earthquake and the subsequent remobilization of the extrados faults, causing accelerated degassing for at least five years.

The case of Medolla illustrates a mechanism of progressive loss of underground fluid pressure, ultimately motivated by earthquake activity but not restricted to the coseismic phase. The leakage occurs along a vertical conduit, as observed also by Gartrell et al.^26^, as a result of the reactivation of smaller extrados faults by the underlying master and seismogenic fault.

Based on the number of wells that have been unsuccessfully drilled in the area one should conclude that the geological setting of the area is fully compatible with the existence of an effective hydrocarbon reservoir associated with the Mirandola anticline, but also that the integrity of this reservoir is long gone.

### Seismic hazard implications

Our dataset shows that in a fold and thrust hydrocarbon province the lack of productive gas reservoirs is likely to be controlled by seismogenic faulting. Conversely, the presence of significant productive reservoirs is in itself an indication of a predominantly aseismic behaviour of the underlying faults; in other words, the ability of a thrust fault to generate significant earthquakes depends primarily on the rheology of the basin deposits. Their behaviour ultimately controls the fate of the reservoirs formed at the core of the largest anticlines; favouring their integrity by promoting aseismic slip over the underlying fault, or causing gas to be lost to the atmosphere by promoting stick–slip. Alternatively, the presence of small- to mid-size reservoirs could be compatible with the presence of faults displaying stick–slip behaviour but capable of smaller earthquakes (< M_w_ 5.5), i.e. faults whose width and slip-per-event are too small to jeopardise the reservoir integrity.

These results are consistent with the observations of Carafa et al.^7^, who showed that the seismic moment released by Italian contractional domains is about 50% of the total moment expected based on fault geometries and slip rates. Such conclusions may imply that Italian thrust faults alternate seismic and aseismic phases in equal proportions, but our findings suggest that this is an unlikely scenario. If a reservoir is unproductive, this is not necessarily an indication that the underlying fault is active, and even less so, that it may generate significant earthquakes, but the reciprocal still holds: if the reservoir is (or has been, before exploitation) fully productive, its controlling fault may be active but is very unlikely to be seismogenic, which is what counts in any seismic hazard analysis. If no other indication of aseismic behaviour is available, for instance from GPS, this evidence alone could justify a significant seismic hazard reduction over thrust faulting systems that exhibit little or no long-term seismicity.

### Worldwide analogs

Our hypotheses are qualitatively supported by other hydrocarbon-bearing fold and thrust belts developed at convergent plate boundaries worldwide (see Figure 2 of Cooper^37^, and Figure 1 of Goffey et al.^38^); they contain 14% of the global known reserves, half of which (49%) lie beneath the Zagros fold belt of Iran, Iraq, and Turkey^37^. We selected three specific actively deforming areas that share a comparable geological setting with our study area and reaveal, even at a rather low resolution, that an anticorrelation between the largest earthquakes and the location of gas fields does exist: the Aquitaine Basin (southwestern France), the Zagros-Persian Gulf area (southern Iran), and the Bengal Basin (Bangladesh–India boundary).

#### Aquitaine basin

This historical hydrocarbon province is located in the southwest of France, covering an area of about 35,000 km^2^ (see Figure 1 in Bahnan et al.^39^). It comprises several geological domains, including the Pyrenean active fold and thrust belt and its foreland, formed as a consequence of the subduction of the Iberian plate beneath the European plate^40^. To the north, the leading edge of the fold and thrust belt hosts the most important hydrocarbon field of metropolitan France, the Lacq gas field^41^. Gas was confined in a structural trap within the pre-Aptian carbonate sequence located in an anticline at a depth of 3500–4050 m, below the oil field, located at 650–700 m depth. Gas was exploited until 2013, when the field became one of the most important pilot sites for Carbon Capture and Storage (CCS^42^).

#### Zagros fold and thrust belt

This large belt forms the external portion of the Zagros active orogenic wedge. It is the most recent result of the convergence and closure of the Neo-Tethys oceanic domain between Arabia and Eurasia^43^. In the Zagros fold and thrust belt the Arabian passive margin sequence has been decoupled from its basement and deformed by large-scale folding and thrusting. A rich seismicity record indicates that within the underlying Panafrican basement, shortening is presently accommodated by reverse faulting^5,44,45^.

The Zagros fold and thrust belt comprises one of the most prolific hydrocarbon provinces worldwide (see map by Esrafili-Dizaji and Rabbani^46^). Most of the gas fields are concentrated in the Fars region and in the contiguous offshore area and accumulated in the Permo-Triassic carbonates, within the Kangan and Dalan formations, the most important carbonate reservoir rocks in the southwestern part of Iran^47,48^. Gas reservoirs lie at depths ranging from 2600 to 3600 m (e.g. Salman gas field^48^).

#### Bengal basin

This large, highly productive and still largely unexplored hydrocarbon province lies on the eastern side of the Indian subcontinent, between the Shillong Plateau to the north, and the Indo-Burman Ranges to the east, in the Tripura district (e.g. Figure 2 in Brahma et al.^49^). This basin originated during the collision of India with Eurasia and Burma, building the extensive Himalayan and Indo-Burman mountain ranges, and thereby loading the lithosphere to form flanking sedimentary basins^50^. The Bengal Basin is a prolific petroleum-bearing basin where 25 economically-viable fields provide about 18% of India's known reserves. It contains up to 22,000 m of Cretaceous to Holocene sedimentary fill^51^, which includes 4000–5000 m of hydrocarbon-bearing Surma Group sandstones^52^, deposited as a consequence of major Miocene uplift in the Himalayas which funneled large volumes of sediments into the basin.

Unfortunately, none of these regions features rich, reliable and openly accessible databases such as ViDEPI and DISS. For this reason, our hypotheses here will need to be supported by targeted investigations. In Italy, hydrocarbon data have become available after a 60 years-phase of intense exploitation, and solid earthquake and seismogenic source data are regularly made available by INGV through an agreement with the Italian Civil Protection Department. Yet, the lesson learned from Italian data may be useful for scientists and hydrocarbon industry practitioners who are puzzled by the relations between the exploitation of gas reservoirs and the occurrence of significant earthquakes.

Understanding the relationships between gas-dominated hydrocarbon fields and seismogenic sources has crucial implications for three independent issues of great societal relevance: (a) supporting the decision-making in the exploitation of as yet unexplored gas pools; (b) offering crucial insight into the seismic hazard of areas undergoing active crustal shortening, and specifically on the seismic/aseismic ratio of the local strain release; and (c) providing guidelines for the identification of suitable underground energy and CO_2_ storage sites, which represent one of the viable options for a gradual transition from fossil fuels to a carbon–neutral economy^53^. Our findings indicate that the best option in planning such facilities is to stay away from large seismogenic faults and opt for a depleted reservoir, as choosing a reservoir whose past performance is unknown would greatly increase the risk of dispersion of CH_4_ (or CO_2_) to the atmosphere. In fact, all existing Italian gas storage sites run by Stogit-SNAM and by Edison Stoccaggio SpA totally leave out the Rimini-Ancona stretch (white rectangle in Fig. 1), the highest seismic hazard portion of our study area.

## Methods

To investigate any spatial correlation between the location of productive gas fields and the occurrence of strong earthquakes (M_w_ 5.5 +) in our study area (Fig. 1) we adopted the Weight of Evidence (WofE) method, a bivariate statistical analysis based on the Bayesian probability framework^22^. The data considered in the analysis were collected from two public-domain datasets: the Database of Individual Seismogenic Sources, built and maintained by INGV (http://diss.rm.ingv.it/diss/), and the “Visibility of Petroleum Exploration Data in Italy” (ViDEPI) database of borehole and geophysical data, assembled by the Italian Ministry of Economic Development (https://www.videpi.com/videpi/pozzi/pozzi.asp).

### The Database of Individual Seismogenic Sources (DISS)

DISS is an original georeferenced repository of tectonic, fault, and paleo-seismological information for the whole of Italy and its surrounding countries and seas. It was conceived in the late 1990s as a tool devoted to supporting seismic hazard assessment in Italy^54^. After its first publication in 2001^55^ it has been revised and updated several times. The most recent version was published in 2021 (v. 3.3.0^56^).

The DISS contains information on seismogenic faults located in the Italian peninsula and the surrounding regions. The information is supplied through two main categories of seismogenic sources^54^: Individual (ISSs), and Composite (CSSs). The ISSs are simplified (rectangular) 3D representations of seismogenic fault planes, generally associated with significant known earthquakes. For each ISS the database provides a full set of geometric (strike, dip, length, width, and depth), kinematic (rake) and seismological parameters (single event displacement, slip rate, recurrence interval), including the expected maximum earthquake size. In contrast, the CSSs are simplified 3D representations of crustal fault systems that are not segmented nor associated with one or more specific earthquakes: each CSS may hence contain an unknown number of ISSs. For this study we considered the eighteen ISSs falling within the study area (Fig. 1a), whose parameters (including the most recent earthquakes that can be associated with each of them) are summarised in Table 1. All ISS are also shown using a 3D perspective in Fig. 1b, which allows the reader to appreciate the relationships between their position and the location of the productive and unproductive gas wells, considered as proxies for the relevant reservoirs. The CSSs are not used in this work, but are shown in Fig. 1 to highlight the continuity of the main seismogenic trends.

### The ViDEPI database of Italian hydrocarbon data

We carried out a comprehensive analysis of data from 1808 wells, a subset of a large body of information acquired since 1957 by several oil companies and made available by the Italian Ministry of Economic Development within the ViDEPI project (http://www.videpi.com/). Current Italian regulations establish that the oil companies shall provide the Ministry with technical reports on the activities that have been carried out. The data, which were originally available only as hard copies, have been scanned, geo-referenced and distributed free of charge through the project website. The available data consist of composite logs containing the following information: (1) lithology, (2) geological formation name(s), (3) formation(s) age(s), (4) depths, (5) litho-stratigraphy, (6) fluid occurrence, (7) depositional environment of each formation, (8) biostratigraphy, and (9) geophysical logs. Pressure and temperature values are sometimes reported, but little or no information is provided concerning the actual exploitation history of the hydrocarbon.

We analysed in detail each well falling within the study area, gathering the parameters needed for our investigation and for assessing their reliability based on the quality of accompanying information. We first removed 49 poorly documented wells, 59 shallow gas wells (< 500 m) and 49 dominantly oil wells, as they do not provide relevant information for our scopes, thereby focusing exclusively on deeper gas wells. We then subdivided the remaining 1651 wells into two categories: (1) positively *unproductive*, when they encountered no exploitable hydrocarbons, and (2) positively *productive*, when they did encounter hydrocarbons, regardless of whether they have been or are currently being exploited.

### Building a model for the GIS analysis

Information regarding the selected wells was loaded to ArcGis along with the 18 selected Individual Seismogenic Sources from the DISS database (out of a total of 127 ISSs in DISS' current version: Fig. 1a). Thanks to the information provided by the ViDEPI, all wells were first projected down to their actual maximum depth, then classified based on the criteria delineated above and on the depth of the exploited hydrocarbon pool (Fig. 1b).

### Weight of Evidence (WofE) method

This method consists of a bivariate statistical analysis based on the Bayesian probability framework^22^. It allows the association of potential evidence in support of a given hypothesis and is easy to run within common GIS software packages. We adopted it to evaluate the spatial relationships between the distribution of productive or unproductive gas wells and the location of major seismogenic faults.

The WofE method is commonly used in the geostatistic field, for example for identifying potentially productive mining areas, locating flowing wells, or mapping cliff instabilities associated with mine subsidence^57^. Recently it was used also for assessing landslide susceptibility^58–60^. The computational framework of the method^22,57^ is briefly summarised below.

The method estimates the weight for evidence (W), which can be positive or negative:

1 $${\text{W}}^{ + } = ln\frac{{{\text{P}}\left\{ {\text{F|E}} \right\}}}{{{\text{P}}\left\{ {{\text{F|}}}\overline{\text{E}} \right\}}}$$

2 $${\text{W}}^{ - } = ln\frac{{{\text{P}}\left\{ \overline{\text{F}}{{\text{|E}}} \right\}}}{{{\text{P}}\left\{ {{{\overline{\text{F}}|\overline{\text{E}}}}} \right\}}}$$

where P{F|*E*} and ${\text{P}}\left\{ {{\text{F|}}}\overline{\text{E}} \right\}$ are the conditional probabilities of being and not being within the factor F, given the presence or the absence of the event E. ${\text{P}}\left\{ \overline{\text{F}}{{\text{|E}}} \right\}$ and ${\text{P}}\left\{ {{{\overline{\text{F}}|\overline{\text{E}}}}} \right\}$ indicate the conditional probability of not being within the factor F, given the presence or the absence of the event E.

The difference between the positive and negative weights is defined as the weight contrast C (= W+ − W−), which provides a measure of the spatial correlation between a certain factor and the occurrence of an event (see Tables 2, S2, and Figs. 2, S2). A negative C indicates the absence of a factor where an event occurs, whereas a C around zero implies no significant relationship with the occurrence of the event.

The statistical significance of the weights and of C can be estimated by considering their standard deviation and variances as:

3 $${\text{S}}\left( {\text{C}} \right) = \sqrt {{\text{S}}_{{{\text{W}} + }}^{2} + {\text{S}}_{{{\text{W}} - }}^{2} }$$

4 $${\text{S}}_{{{\text{W}}^{+}}}^{2} = \frac{1}{{{\text{P}}\left\{ {\text{F|E}} \right\}}} + \frac{1}{{{\text{P}}\left\{ {{\text{F|}}}\overline{\text{E}} \right\}}}$$

5 $${\text{S}}_{{{\text{W}}^{-}}}^{2} = \frac{1}{{{\text{P}}\left\{ \overline{\text{F}}{{\text{|E}}} \right\}}} + \frac{1}{{{\text{P}}\left\{ {{{\overline{\text{F}}|\overline{\text{E}}}}} \right\}}}$$

where S^2^_W+_ and S^2^_W−_ are the variance of W^+^ and W^−^, respectively^61^. Finally, the Studentised Contrast C/S(C) provides an assessment of the quality of the results.

To estimate the conditional probabilities of Eqs. (1) and (2) we first discretised the entire study area with a regular 10 × 10 m grid comprising 31,541 × 39,330 pixels (lines × columns). We then calculated the distance of each pixel to the nearest seismogenic fault, following two strategies: in 2D (planar), or in full 3D, which we consider more appropriate for investigating the motivations of the statistical evidence. These distances correspond to the factor F in Eqs. (1) and (2).

For the 3D case, we calculated the shortest distance from the bottom of each well to the surface of the nearest seismogenic fault plane. In contrast, for the 2D case we calculated the shortest distance between the head of each well and a point along the external boundary of a 2 km-wide buffer area encircling the surface projection of the nearest seismogenic fault. All calculated distances were then grouped into eight bins: 0–2, 2–5, 5–10, 10–20, 20–30, 30–40, 40–50, 10–50 and > 50 km for the 2D case; and 0–4, 4–6, 6–10, 10–20, 20–30, 30–40, 40–50, 10–50, > 50 km for the 3D case, respectively. Notice that the width of the distance bins was decided based on a series of trial-and-error tests, aimed at exploring any possible bias in the results due to the choice of the bin size. We maintain that the selected bin widths allow for a fine sampling of well-fault distances for the shorter distance values, which is crucial for any geological and geophysical interpretation of the results, without generating any undesired bias. Finally, each pixel was assigned to a group, depending on the above classification. Tables S1–S2 and Figs. S1–S2 show the distribution of gas wells and the spatial distribution of the different classes of pixels with respect to the binning scheme (Fig. S2 shows the 3D case only).

Based on Eqs. (1) and (2), the weights of evidence can be modified (when analysing one group at a time) in numbers of pixels, as in the following:

6 $$W_{i}^{ + } = \ln \frac{{\frac{{N{\text{pix}}_{{1}} }}{{N{\text{pix}}_{{1}} + N{\text{pix}}_{{2}} }}}}{{\frac{{N{\text{pix}}_{{3}} }}{{N{\text{pix}}_{{3}} + N{\text{pix}}_{{4}} }}}}\quad W_{i}^{ - } = \ln \frac{{\frac{{N{\text{pix}}_{{2}} }}{{N{\text{pix}}_{{1}} + N{\text{pix}}_{{2}} }}}}{{\frac{{N{\text{pix}}_{{4}} }}{{N{\text{pix}}_{{3}} + N{\text{pix}}_{{4}} }}}}$$

where *N*pix_1_ is the number of pixels—that is to say, wells—falling within the considered distance bin (e.g., the 0–2 km interval); *N*pix_2_ is the number of pixels that correspond to a gas well (either productive or unproductive) but fall outside the considered distance bin; *N*pix_3_ is the number of pixels related to a potential event predictive factor (selected group of distance) but without any wells; and *N*pix_4_ is the number of pixels where neither the considered potential event predictive factor nor a well are observed.

The predictive capabilities of the proposed model were checked through a cross-validation procedure which consists in subdividing a data sample into two subsets^62^. The analysis is then performed on one subset, referred to as *training dataset*, which includes 60% of the wells, randomly selected from the whole available sample, while the validation is carried out on the remaining 40% of the data, referred to as *test dataset*. In order to cover the entirety of the *training*/*validation* data subsets, this random procedure was repeated ten times for the 2D case and 10 times for the 3D case: Table S3 summarises the variability of the C obtained for the ten tests and the associated standard deviation, which are encourangingly low.

### Area under the curve test

The validity and the accuracy of the C distributions were tested using the *success-rate* and *prediction-rate* curves in combination with the Area Under the Curve (AUC): a method that provides a measure of the total accuracy based on the rate curves, where a total area equal to one indicates perfect accuracy^63^ (Fig. 3). The success-rate curve, which is based on the *training dataset*, shows how good the selected parameter curves are in fitting the known wells (productive or unproductive). The prediction rate curve, which is based on the *testing dataset*, provides quantitative information for forecasting the position of a productive or unproductive well. The most common procedure involves sorting in descending order the calculated index values that refer to the total number of cells in the study area.

### Moran’s Index tests

Finally, we carried out both global and local Moran’s Index tests, highlighting (a) that the dataset is clustered, and (b) that the unproductive and productive wells cluster differently as their location relative to the seismogenic faults changes (see Figs. S3–S6).

## Supplementary Information

Supplementary Information.

## Data Availability

All hydrocarbon data used in this work come from the ViDEPI project database (https://www.videpi.com/videpi/videpi.asp). All seismogenic source data were obtained from DISS, the Database of Individual Seismogenic Sources, v. 3.3.0 (http://diss.rm.ingv.it/diss/). The underground storage facilities operated by Stogit-SNAM SpA are available at https://www.snam.it/en/about-us/geographical-presence/index.html. The underground storage facilities operated by Edison Stoccaggio SpA are available at https://www.edisonstoccaggio.it/en/activities-and-facilities/our-plants/. All of these sites were last accessed on 20 January 2022.
