# Supplementary material for: Gas fields and large shallow seismogenic reverse faults are anticorrelated
Source: Sci Rep. 2022 Feb 3;12:1827. doi: 10.1038/s41598-022-05732-8 (PMC8814180; doi:10.1038/s41598-022-05732-8)
Supplement: Supplementary file 1 — Supplementary Information. [file 41598_2022_5732_MOESM1_ESM.docx]

**Gas fields and large shallow seismogenic reverse faults are anticorrelated**

**Supplementary Information**

This section supplies information on the distribution of well-seismogenic fault distances used in the study, on the calculation of the parameters of the Weight of Evidence test and on the calculation of the global and local Moran's Index that was adopted to validate the analyses conducted.

**Table S1: Distribution of seismogenic fault-well distances for the 2D and 3D scenarios.**

| **Distance bins 2D (km)** | **Productive** | **Unproductive** |
| --- | --- | --- |
| 0-2 | 21 | 92 |
| 2-5 | 8 | 53 |
| 5-10 | 69 | 91 |
| 10-20 | 219 | 247 |
| 20-30 | 215 | 150 |
| 30-40 | 141 | 79 |
| 40-50 | 102 | 39 |
| >50 | 56 | 69 |
| **Grand total** | **831** | **820** |

| **Distance bins 3D (km)** | **Productive** | **Unproductive** |
| --- | --- | --- |
| 0-4 | 18 | 39 |
| 4-6 | 11 | 73 |
| 6-10 | 36 | 90 |
| 10-20 | 230 | 268 |
| 20-30 | 231 | 158 |
| 30-40 | 145 | 81 |
| 40-50 | 104 | 42 |
| 50-150 | 56 | 69 |
| **Grand total** | **831** | **820** |

Summary of the the seismogenic fault-well distances falling withing each bin (see also the histogram in Figure S1).­

**
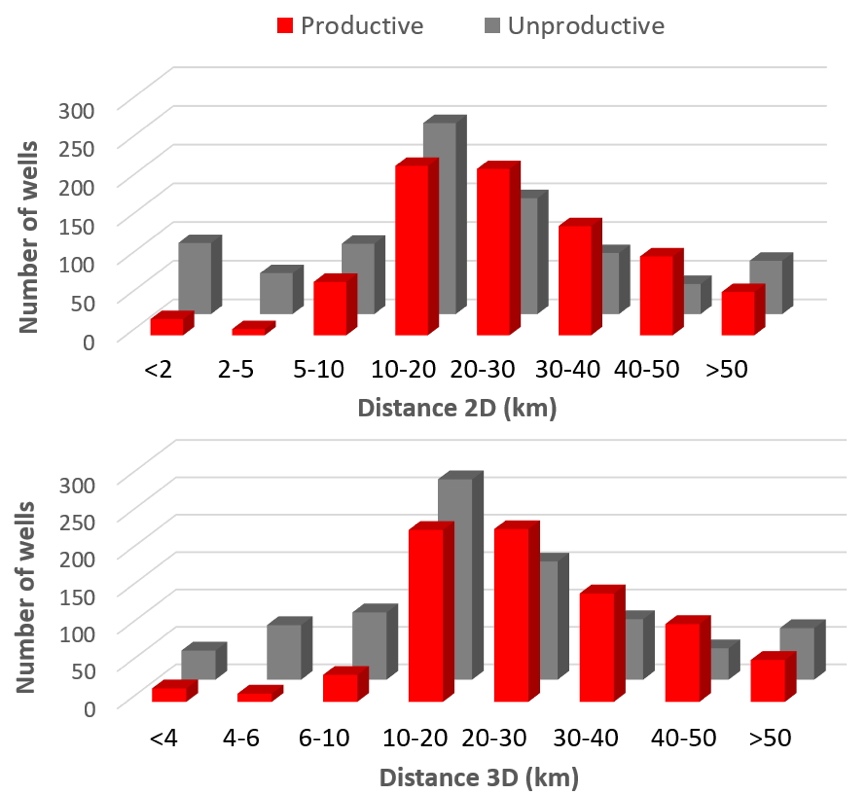
**

**Figure S1: Histograms of seismogenic fault-well distances for the 2D and 3D scenarios.**

Histograms of the seismogenic fault-well distances grouped in the bins used for the WofE analysis (see Table S1). The image provides a visual confirmation of the sharp predominance of unproductive gas wells with respect to productive ones within 10 km of the seismogenic faults adopted in our study.

**Table S2: Summary of the results obtained by the Weight of Evidence test.**

a)

| **2D well-fault buffer distance (km)** | **Total**  **cells** | **Cells hosting productive**  **wells** | **% of total wells** | **Cells NOT hosting productive**  **wells** | **W+** | **S^2^_W-_** | **W-** | **S^2^_W+_** | **C** | **C/S(C)** |
| --- | --- | --- | --- | --- | --- | --- | --- | --- | --- | --- |
| <2 | 18,827,023 | 14 | 2.8 | 18,827,009 | -0.363 | 0.071 | 0.013 | 0.002 | -0.375 | -1.385 |
| 2-5 | 21,451,808 | 3 | 0.6 | 21,451,805 | -2.034 | 0.333 | 0.041 | 0.002 | -2.075 | -3.583 |
| 5-10 | 39,929,503 | 50 | 10.0 | 39,929,453 | 0.158 | 0.020 | -0.016 | 0.002 | 0.175 | 1.172 |
| 10-20 | 85,400,755 | 133 | 26.7 | 85,400,622 | 0.377 | 0.020 | -0.108 | 0.002 | 0.485 | 4.786 |
| 20-30 | 63,252,541 | 120 | 24.0 | 63,252,421 | 0.574 | 0.020 | -0.130 | 0.002 | 0.703 | 6.715 |
| 30-40 | 52,505,078 | 88 | 17.6 | 52,504,990 | 0.450 | 0.020 | -0.075 | 0.002 | 0.525 | 4.467 |
| 40-50 | 43,392,493 | 61 | 12.2 | 43,392,432 | 0.274 | 0.016 | -0.033 | 0.002 | 0.307 | 2.246 |
| > 50 | 142,165,247 | 30 | 6.0 | 14,2165,217 | -1.622 | 0.033 | 0.301 | 0.002 | -1.923 | -10.213 |
| ***Grand total*** | ***466,924,448*** | ***499*** | ***100.0*** |  |  |  |  |  |  |  |
|  |  |  |  |  |  |  |  |  |  |  |
| **2D well-fault buffer distance (km)** | **Total**  **cells** | **Cells hosting unproductive**  **wells** | **% of total wells** | **Cells NOT hosting unproductive**  **wells** | **W+** | **S^2^_W-_** | **W-** | **S^2^_W+_** | **C** | **C/S(C)** |
| <2 | 18,827,023 | 62 | 12.6 | 188,26,961 | 1.140 | 0.016 | -0.094 | 0.002 | 1.233 | 9.077 |
| 2-5 | 21,451,808 | 31 | 6.3 | 21,451,777 | 0.316 | 0.032 | -0.018 | 0.002 | 0.334 | 1.800 |
| 5-10 | 39,929,503 | 52 | 10.6 | 39,929,451 | 0.212 | 0.019 | -0.022 | 0.002 | 0.234 | 1.597 |
| 10-20 | 85,400,755 | 151 | 30.7 | 85,400,604 | 0.518 | 0.019 | -0.165 | 0.002 | 0.682 | 6.979 |
| 20-30 | 63,252,541 | 91 | 18.5 | 63,252,450 | 0.311 | 0.019 | -0.059 | 0.002 | 0.370 | 3.190 |
| 30-40 | 52,505,078 | 45 | 9.1 | 52,505,033 | -0.207 | 0.019 | 0.023 | 0.002 | -0.230 | -1.470 |
| 40-50 | 43,392,493 | 23 | 4.7 | 43,392,470 | -0.687 | 0.043 | 0.050 | 0.002 | -0.737 | -3.450 |
| > 50 | 142,165,247 | 37 | 7.5 | 14,2165,210 | -1.398 | 0.027 | 0.285 | 0.002 | -1.683 | -9.847 |
| ***Grand total*** | ***466,924,448*** | ***492*** | ***100.0*** |  |  |  |  |  |  |  |

b)

| **3D well-fault distance (km)** | **Total**  **cells** | **Cells hosting productive**  **wells** | **% of total wells** | **Cells NOT hosting productive**  **wells** | **W+** | **S^2^_W-_** | **W-** | **S^2^_W+_** | **C** | **C/S(C)** |
| --- | --- | --- | --- | --- | --- | --- | --- | --- | --- | --- |
| <4 | 8,840,000 | 10 | 2.0 | 8,839,990 | 0.057 | 0.100 | -0.001 | 0.002 | 0.058 | 0.180 |
| 4-6 | 20,330,000 | 7 | 1.4 | 20,329,993 | -1.133 | 0.143 | 0.030 | 0.002 | -1.163 | -3.060 |
| 6-10 | 40,000,000 | 27 | 5.4 | 39,999,973 | -0.459 | 0.037 | 0.034 | 0.002 | -0.493 | -2.494 |
| 10-20 | 92,830,000 | 143 | 28.7 | 92,829,857 | 0.366 | 0.037 | -0.116 | 0.002 | 0.482 | 4.865 |
| 20-30 | 67,170,000 | 128 | 25.7 | 67,169,872 | 0.578 | 0.037 | -0.141 | 0.002 | 0.719 | 7.018 |
| 30-40 | 54,260,000 | 92 | 18.4 | 54,259,908 | 0.462 | 0.037 | -0.080 | 0.002 | 0.542 | 4.694 |
| 40-50 | 44,190,000 | 63 | 12.6 | 44,189,937 | 0.288 | 0.016 | -0.036 | 0.002 | 0.324 | 2.402 |
| >50 | 139,304,448 | 29 | 5.8 | 139,304,419 | -1.636 | 0.034 | 0.294 | 0.002 | -1.930 | -10.088 |
| ***Grand total*** | ***466,924,448*** | ***499*** | ***100.0*** |  |  |  |  |  |  |  |
|  |  |  |  |  |  |  |  |  |  |  |
| **3D well-fault distance (km)** | **Total**  **cells** | **Cells hosting unproductive**  **wells** | **% of total wells** | **Cells NOT hosting unproductive**  **wells** | **W+** | **S^2^_W-_** | **W-** | **S^2^_W+_** | **C** | **C/S(C)** |
| <4 | 8,840,000 | 29 | 5.9 | 8,839,971 | 1.136 | 0.034 | -0.042 | 0.002 | 1.177 | 6.150 |
| 4-6 | 20,330,000 | 48 | 9.8 | 20,329,952 | 0.807 | 0.021 | -0.058 | 0.002 | 0.865 | 5.693 |
| 6-10 | 40,000,000 | 45 | 9.2 | 39,999,955 | 0.065 | 0.022 | -0.006 | 0.002 | 0.072 | 0.459 |
| 10-20 | 92,830,000 | 167 | 33.8 | 92,829,833 | 0.535 | 0.022 | -0.193 | 0.002 | 0.728 | 7.645 |
| 20-30 | 67,170,000 | 95 | 19.3 | 67,169,905 | 0.294 | 0.022 | -0.059 | 0.002 | 0.354 | 3.096 |
| 30-40 | 54,260,000 | 46 | 9.4 | 54,259,954 | -0.217 | 0.022 | 0.025 | 0.002 | -0.243 | -1.568 |
| 40-50 | 44,190,000 | 25 | 5.1 | 44,189,975 | -0.622 | 0.040 | 0.047 | 0.002 | -0.669 | -3.260 |
| >50 | 139,304,448 | 37 | 7.5 | 139,304,411 | -1.378 | 0.027 | 0.276 | 0.002 | -1.654 | -9.676 |
| ***Grand total*** | ***466,924,448*** | ***492*** | ***100.0*** |  |  |  |  |  |  |  |

The table provides an overview of the input dataset and of all results obtained by the Weight of Evidence test for 2D (a) and 3D distances (b), respectively, for both productive and unproductive wells.

Notice that the ratio between the contrast weight and the standard deviation for the closest bins in the productive well analysis is close to 1 for the 2D well-seismogenic fault buffer distance, indicating a relatively low significance of the absolute estimated value. Nevertheless, the C values range from nearly zero to negative values: this in itself indicates a non-correlation or a negative correlation between productive wells and seismogenic faults. The ratio between the contrast weight and the standard deviation for the closest bin of the 3D distance scenario indicates that an interpretation of the results should not be attempted.

**Figure S2: Geographical distribution of productive and unproductive wells in the eight bins selected for the 3D fault-well distance scheme.**

| 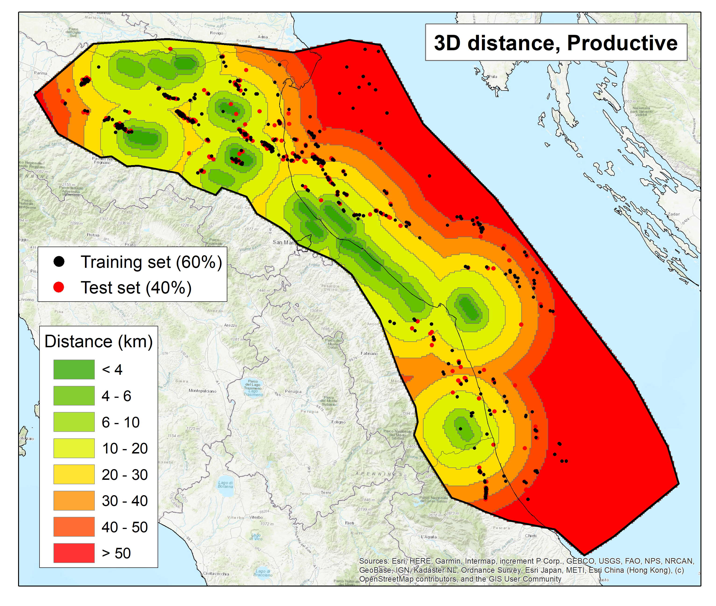 | 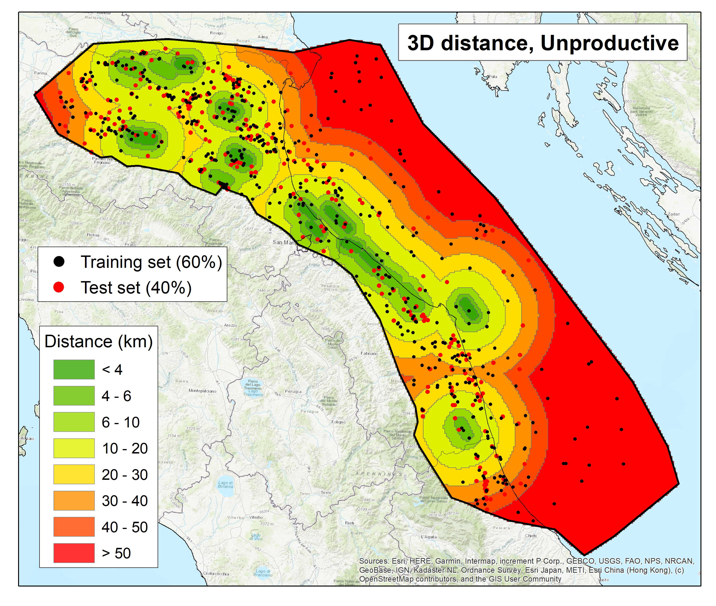 |
| --- | --- |

Geographical distribution of gas wells and of the different classes of pixels with respect to the distance binning scheme (for the 3D case only). The figure was drawn using ArcGis 10.4.1 for Desktop/ArcMap 10.4.1.5686.

**Table S3: Summary of the C value obtained by the Weight of Evidence test for 10 randomly sampled models.**

| **Productive** | | | | | | | | | | | | |
| --- | --- | --- | --- | --- | --- | --- | --- | --- | --- | --- | --- | --- |
| 2D Distance (km) | **M1** | M2 | M3 | M4 | M5 | M6 | M7 | M8 | M9 | M10 | Mean | StdDev |
| <2 | **-0.375** | -0.375 | -0.304 | -0.375 | -0.720 | -0.623 | -0.947 | -0.452 | -0.375 | -0.623 | -0.517 | 0.195 |
| 2-5 | **-2.075** | -2.075 | -2.075 | -1.375 | -1.560 | -1.785 | -2.075 | -1.560 | -1.375 | -1.375 | -1.733 | 0.302 |
| 5-10 | **0.175** | -0.098 | 0.034 | -0.071 | -0.017 | 0.058 | 0.058 | -0.246 | -0.126 | -0.017 | -0.025 | 0.111 |
| 10-20 | **0.485** | 0.474 | 0.412 | 0.485 | 0.535 | 0.443 | 0.454 | 0.545 | 0.495 | 0.433 | 0.476 | 0.041 |
| 20-30 | **0.703** | 0.670 | 0.758 | 0.659 | 0.625 | 0.725 | 0.768 | 0.810 | 0.841 | 0.862 | 0.742 | 0.076 |
| 30-40 | **0.525** | 0.552 | 0.483 | 0.497 | 0.538 | 0.566 | 0.483 | 0.511 | 0.469 | 0.440 | 0.506 | 0.038 |
| 40-50 | **0.307** | 0.449 | 0.380 | 0.449 | 0.432 | 0.326 | 0.432 | 0.210 | 0.269 | 0.326 | 0.358 | 0.079 |
| >50 | **-1.923** | -1.758 | -1.728 | -1.758 | -1.790 | -1.758 | -1.790 | -1.758 | -1.888 | -1.822 | -1.797 | 0.060 |
| 3D Distance (km) | **M1** | M2 | M3 | M4 | M5 | M6 | M7 | M8 | M9 | M10 | Mean | StdDev |
| <4 | **0.058** | 0.155 | 0.155 | 0.155 | -0.049 | -0.049 | -0.645 | 0.155 | 0.155 | 0.155 | 0.025 | 0.237 |
| 4-6 | **-1.163** | -1.319 | -1.163 | -0.908 | -1.319 | -1.319 | -1.319 | -1.163 | -0.908 | -1.319 | -1.190 | 0.157 |
| 6-10 | **-0.493** | -0.808 | -0.618 | -0.757 | -0.662 | -0.618 | -0.757 | -0.808 | -0.757 | -0.709 | -0.699 | 0.095 |
| 10-20 | **0.482** | 0.422 | 0.402 | 0.422 | 0.501 | 0.432 | 0.472 | 0.442 | 0.432 | 0.402 | 0.441 | 0.032 |
| 20-30 | **0.719** | 0.698 | 0.771 | 0.709 | 0.666 | 0.740 | 0.792 | 0.832 | 0.862 | 0.891 | 0.768 | 0.071 |
| 30-40 | **0.542** | 0.555 | 0.488 | 0.515 | 0.501 | 0.568 | 0.488 | 0.528 | 0.460 | 0.417 | 0.506 | 0.043 |
| 40-50 | **0.324** | 0.462 | 0.360 | 0.429 | 0.462 | 0.342 | 0.412 | 0.210 | 0.268 | 0.342 | 0.361 | 0.078 |
| >50 | **-1.930** | -1.729 | -1.699 | -1.729 | -1.792 | -1.760 | -1.760 | -1.729 | -1.825 | -1.792 | -1.775 | 0.063 |
| **Unproductive** | | | | | | | | | | | | |
| 2D Distance (km) | **M1** | M2 | M3 | M4 | M5 | M6 | M7 | M8 | M9 | M10 | Mean | StdDev |
| <2 | **1.233** | 1.269 | 1.117 | 1.076 | 1.157 | 1.157 | 1.097 | 1.177 | 1.076 | 1.056 | 1.142 | 0.067 |
| 2-5 | **0.334** | 0.187 | 0.334 | 0.401 | 0.368 | 0.263 | 0.226 | 0.433 | 0.433 | 0.187 | 0.316 | 0.091 |
| 5-10 | **0.234** | 0.396 | 0.469 | 0.337 | 0.276 | 0.376 | 0.255 | 0.396 | 0.168 | 0.122 | 0.303 | 0.105 |
| 10-20 | **0.682** | 0.555 | 0.565 | 0.605 | 0.614 | 0.555 | 0.614 | 0.524 | 0.663 | 0.739 | 0.612 | 0.063 |
| 20-30 | **0.370** | 0.436 | 0.329 | 0.397 | 0.384 | 0.423 | 0.329 | 0.397 | 0.410 | 0.384 | 0.386 | 0.034 |
| 30-40 | **-0.230** | -0.280 | -0.182 | -0.306 | -0.206 | -0.230 | -0.027 | -0.280 | -0.091 | -0.206 | -0.204 | 0.082 |
| 40-50 | **-0.737** | -0.783 | -0.737 | -0.737 | -0.608 | -0.692 | -0.568 | -0.649 | -0.608 | -0.832 | -0.695 | 0.081 |
| >50 | **-1.683** | -1.545 | -1.495 | -1.422 | -1.599 | -1.495 | -1.520 | -1.520 | -1.743 | -1.376 | -1.540 | 0.105 |
| 3D Distance (km) | **M1** | M2 | M3 | M4 | M5 | M6 | M7 | M8 | M9 | M10 | Mean | StdDev |
| <4 | **1.177** | 1.020 | 0.886 | 1.020 | 1.062 | 0.837 | 0.933 | 1.020 | 0.977 | 0.977 | 0.991 | 0.090 |
| 4-6 | **0.865** | 0.888 | 0.842 | 0.718 | 0.794 | 0.888 | 0.692 | 0.794 | 0.794 | 0.665 | 0.794 | 0.076 |
| 6-10 | **0.072** | 0.295 | 0.375 | 0.335 | 0.274 | 0.211 | 0.166 | 0.431 | 0.120 | 0.120 | 0.240 | 0.115 |
| 10-20 | **0.728** | 0.579 | 0.617 | 0.655 | 0.636 | 0.645 | 0.673 | 0.570 | 0.701 | 0.701 | 0.650 | 0.050 |
| 20-30 | **0.354** | 0.442 | 0.301 | 0.340 | 0.354 | 0.379 | 0.327 | 0.379 | 0.405 | 0.392 | 0.367 | 0.039 |
| 30-40 | **-0.243** | -0.292 | -0.196 | -0.292 | -0.243 | -0.243 | -0.107 | -0.317 | -0.150 | -0.173 | -0.225 | 0.064 |
| 40-50 | **-0.669** | -0.712 | -0.628 | -0.712 | -0.512 | -0.628 | -0.476 | -0.588 | -0.549 | -0.852 | -0.633 | 0.105 |
| >50 | **-1.654** | -1.516 | -1.465 | -1.393 | -1.570 | -1.465 | -1.491 | -1.491 | -1.714 | -1.347 | -1.511 | 0.105 |

The table shows the results of a test conducted by obtaining different realizations of the *training dataset* (through a random selection), and then calculating the mean and standard deviation of the obtained C values. The operation is repeated ten times for the productive wells subset and ten times for the unproductive wells subset, and shows that their standard deviation is consistently very small. Model 1 is the model used for the analyses in our study (same as in Table S2).

**Figure S3: Global Moran's Index test for the 2D distance scenario.**


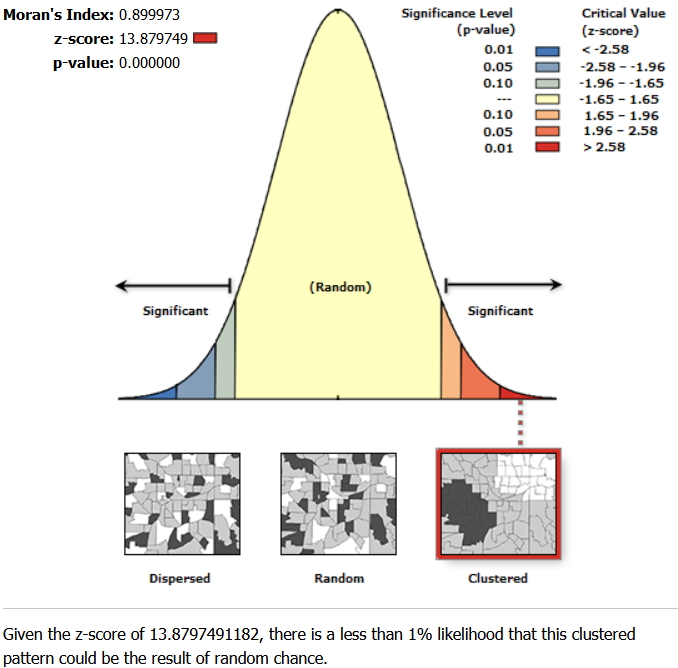


Global Moran's Index test for the 2D distance scenario, obtained using the software ArcGis 10.4.1 for Desktop by by ESRI (<http://www.esri.com>).

The Global Moran’s Index test is used to evaluate the spatial autocorrelation of a set of spatial features, which in our case are the productive/unproductive wells, and of an associated attribute, in our case the 2D/3D distances of all wells from the seismogenic faults. The test evaluates through the *z-score* whether the expressed pattern is clustered, dispersed, or random, but provides only a global, non-geographically distributed answer. The *z-score* calculated for both our distance scenarios is very high, showing that our patterns are highly clustered and very unlikely to result from pure chance.

**Figure S4: Global Moran's Index test for the 3D distance scenario.**


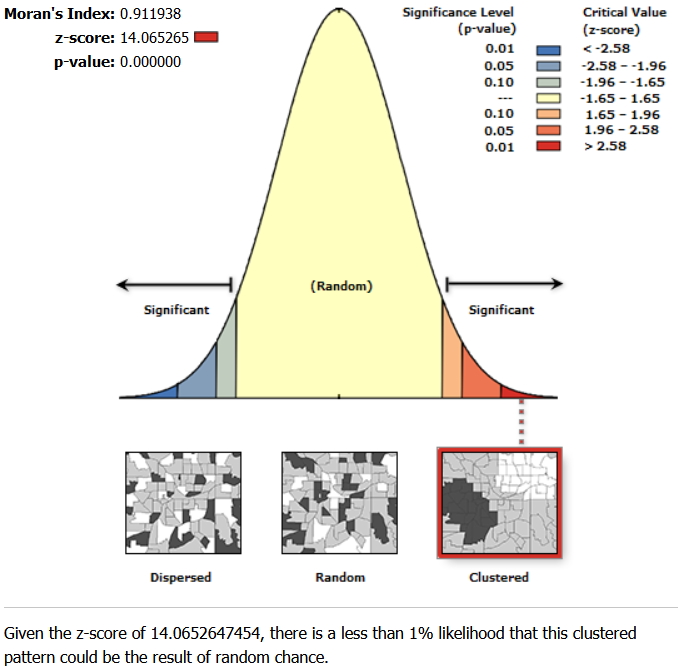


Global Moran's Index test for the 3D distance scenario. obtained using the software ArcGis 10.4.1 for Desktop by by ESRI (<http://www.esri.com>). See the description in the caption of Figure S3.

**Figure S5: Local Moran's Index test for the 2D distance scenario.**

| 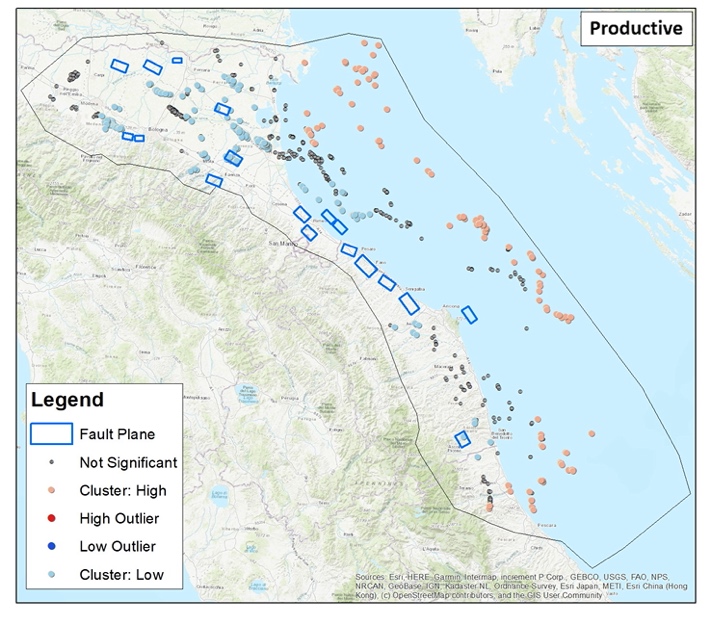 | 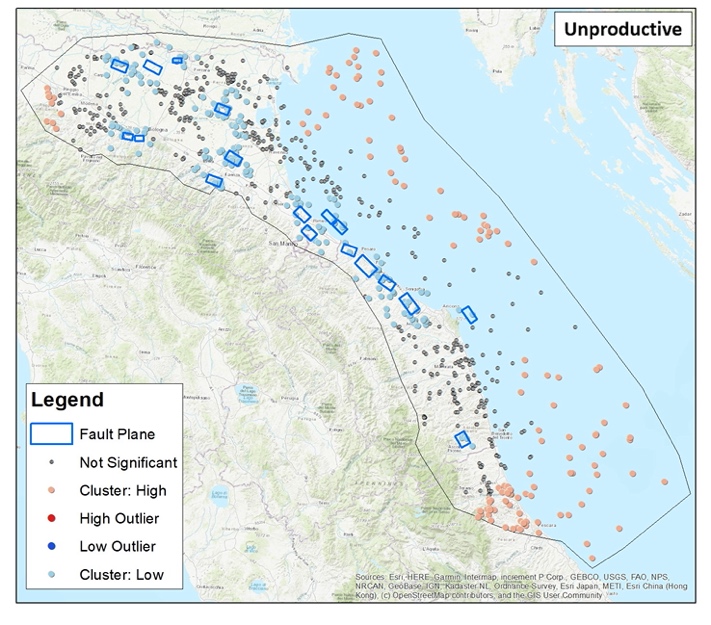 |
| --- | --- |

| **Distance bins 2D (km)** | **Productive** | **Unproductive** |
| --- | --- | --- |
| <2 | 21 | 92 |
| 2-5 | 8 | 49 |
| 5-10 | 65 | 51 |
| 10-20 | 114 | 17 |
| 20-30 | 0 | 0 |
| 30-40 | 10 | 28 |
| 40-50 | 80 | 37 |
| >50 | 56 | 68 |
| **Grand total** | **354** | **342** |


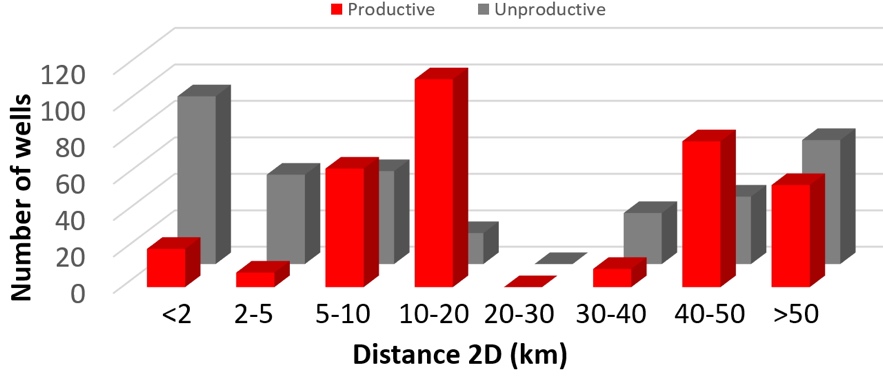


Local Moran's Index test for the 2D distance scenario, obtained using the software ArcGis 10.4.1 for Desktop/ArcMap 10.4.1.5686 by ESRI (<http://www.esri.com>).

The Local Moran's Index test indentifies spatial variations in the distribution of productive/unproductive wells that occur within the study area. The maps show the level of clustering as a function of the position of wells. The histogram shows the distribution of productive/unproductive wells for each distance bin used in the WofE analysis, but only for wells that are considered *clustered* by the Local Moran’s Index test. As expected, the clustering pattern of productive wells is markedly different from that of unproductive wells, thus independently confirming the applicability and the results of the WofE method.

**Figure S6: Local Moran's test for the 3D distance scenario.**

| 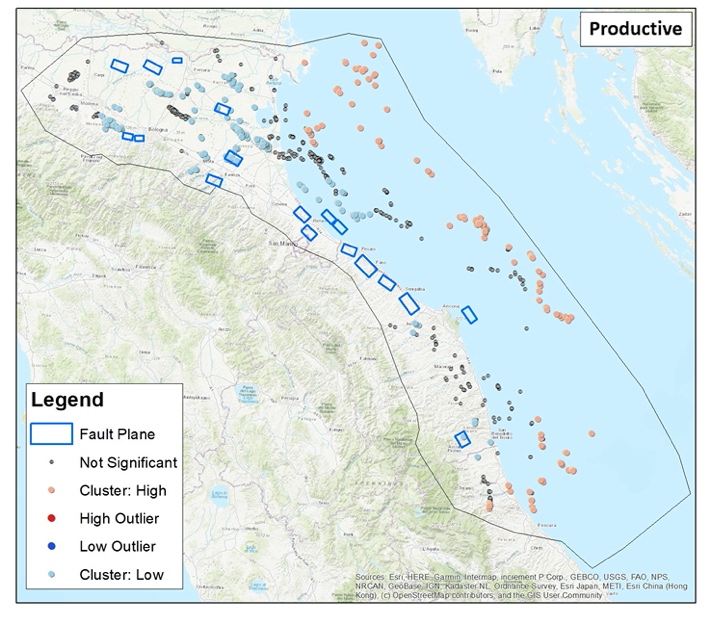 | 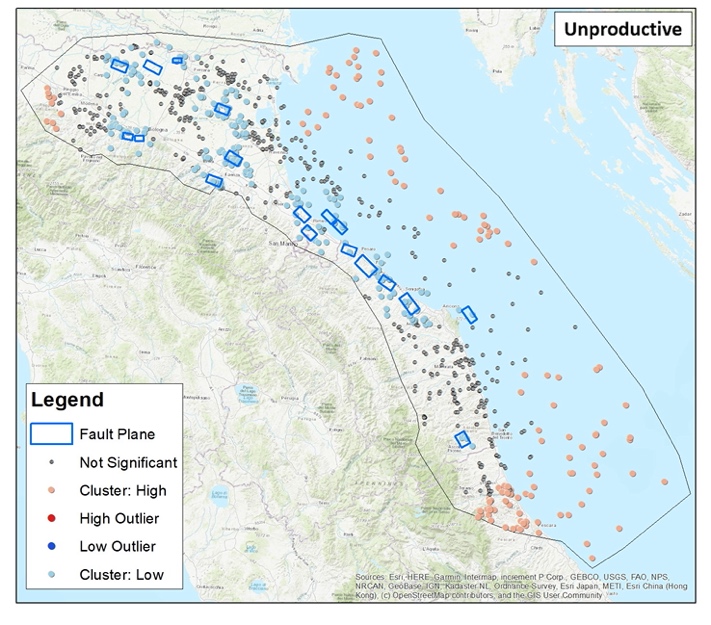 |
| --- | --- |

| **Distance bins 3D (km)** | **Productive** | **Unproductive** |
| --- | --- | --- |
| <4 | 18 | 39 |
| 4-6 | 11 | 69 |
| 6-10 | 34 | 71 |
| 10-20 | 144 | 41 |
| 20-30 | 1 | 0 |
| 30-40 | 11 | 23 |
| 40-50 | 81 | 40 |
| >50 | 56 | 68 |
| **Grand total** | **356** | **351** |


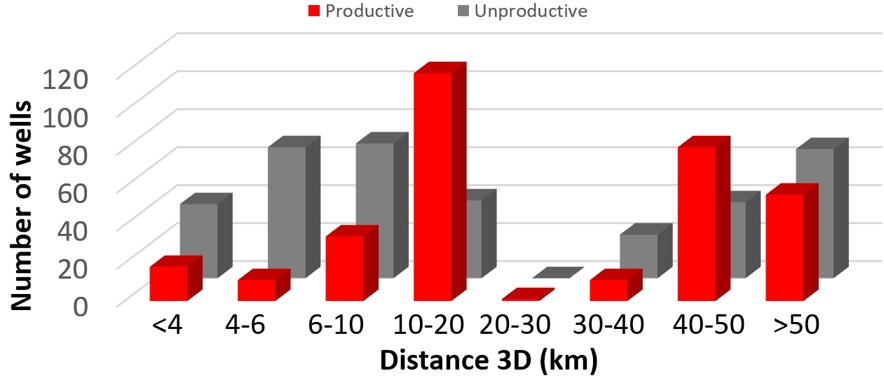


Local Moran's Index test for the 3D distance scenario. The figure was drawn using ArcGis 10.4.1 for Desktop/ArcMap 10.4.1.5686 by ESRI (<http://www.esri.com>). See the description in the caption of Figure S5.­­
